# At-home sample collection is an effective strategy for diagnosis and management of symptomatic and asymptomatic SARS-CoV-2 carriers

**DOI:** 10.1186/s12879-022-07377-4

**Published:** 2022-05-09

**Authors:** Devon P. Humphreys, Kathleen M. Gavin, Kaylan M. Olds, Marc P. Bonaca, Timothy A. Bauer

**Affiliations:** 1 Everly Health, Inc, 823 Congress Ave., Suite 1200, Austin, TX 78701 USA; 2 University of Colorado Anschutz Medical Campus, Aurora, CO USA; 3 CPC Clinical Research, Aurora, CO USA

**Keywords:** SARS-CoV-2, COVID-19, Humans, Adults, Diagnosis, Specimen handling, Prevalence, Risk

## Abstract

**Background:**

Diagnostic testing accessibility and asymptomatic transmission of SARS-CoV-2 present major challenges for curbing and preventing community prevalence of COVID-19. At-home sample collection for molecular testing provides a convenient and effective solution for disease containment and prevention.

**Methods:**

This is a retrospective, cross-sectional, case-control study. Our primary aim was to determine the prevalence and relative risk of SARS-CoV-2 among asymptomatic versus symptomatic individuals using at-home sample collection kits for diagnosis. Participants included adults from across the United States who completed a COVID-19 Home Collection kit between May 2020 and September 2021. Main measurements included self-reported symptoms and at-home self-collected anterior nasal swab RT-PCR test results for SARS-CoV-2.

**Results:**

Data from 282,831 individuals were included in this analysis. The overall SARS-CoV-2 prevalence of at-home test takers was low compared to national averages during this period (3.28% vs. 7.68%). Those reporting no symptoms were at lower risk of positive test results compared to those with symptoms (risk ratio: 0.080, 95% CI, 0.078–0.082). However, of all positive SARS-CoV-2 tests, 48.75% were from individuals reporting no symptoms at the time of testing.

**Conclusions:**

We conclude that at-home sample collection is a viable option and potentially important strategy for improving access to testing, detecting asymptomatic cases, and curbing preventable transmission of COVID-19.

## Introduction

The emergence of the Severe Acute Respiratory Syndrome Coronavirus 2 (SARS-CoV-2) has caused a global health threat, resulting in the rise of a new respiratory illness called Coronavirus Disease 2019 (COVID-19) [1, 2]. Development and implementation of effective testing strategies to detect SARS-CoV-2 infections is critical in the effective containment of COVID-19 and the continuity of economic and educational activities amid this public health crisis. The continued emergence of variants of concern [3] and evidence of transitory immunity and breakthrough cases in those that have been vaccinated [4, 5] highlight the continued importance of public health strategies to help control the spread of the virus.

Nasopharyngeal sampling for Nucleic Acid Amplification Testing (NAAT) is the current noninvasive criterion standard diagnostic test for SARS-CoV-2 [6]. However, it requires in-person sample collection by trained personnel, potentially exposing healthcare workers to infectious aerosols, occupying them from performing other critical tasks, and utilizes limited supplies of personal protective equipment. Fortunately, self-collection options exist, enhancing availability for those in quarantine or with limited access to testing sites. Self-collected samples paired with NAAT testing are valid and well tolerated and are thus an attractive minimally invasive alternative testing methodology with the potential to serve as an important tool to improve community testing access, particularly when conducted at home [7–9].

Many COVID-19 cases present with common symptoms, but asymptomatic cases have challenged social and policy efforts to curb the spread of the virus [10]. Considering that asymptomatic and pre-symptomatic carriers can transmit the virus to others, the presence of asymptomatic individuals carrying SARS-CoV-2 poses a significant public health risk [11–14]. Furthermore, reports that vaccinated individuals are more likely to be asymptomatic when infected, reemphasizes the importance of access to testing regardless of symptoms [15–17] and the value of decentralized testing for transmission prevention [18]. While those experiencing symptoms may choose to seek treatment along with in-person testing, at-home sample collection for asymptomatic individuals may be best practice for testing and management outside of the clinic. An under-reported feature of the SARS-CoV-2 pandemic is the prevalence and relative risk of asymptomatic and symptomatic SARS-CoV-2 infections among at-home test users, which may inform strategies to help lessen the burden on the health care system [18, 19].

The primary objective of this analysis was to estimate the prevalence and relative risk of SARS-CoV-2 infection in asymptomatic and symptomatic individuals from across the United States who used home collection methods. These types of tests combine at-home sample collection with high sensitivity reverse transcriptase polymerase chain reaction (RT-PCR) diagnostic assays to detect SARS-CoV-2. Secondary objectives included characterizing the case positivity rate of SARS-CoV-2 by symptom status and the relative risk of infection among asymptomatic and symptomatic individuals.

## Methods

The current study is a retrospective, cross-sectional, case-control study that was reviewed by WCG IRB (IRB registration no. IRB00000533) and met requirements for a waiver of informed consent under 45 CFR 46.116(f). All methods were carried out in accordance with relevant guidelines and regulations. Data included in this analysis was from at-home collection kits for COVID-19 testing that were voluntarily purchased from Everlywell, Inc.

### Data collection and testing

On May 13, 2020, the United States (U.S.) Food and Drug Administration (FDA) issued an Emergency Use Authorization (EUA) for the Everlywell COVID-19 Test Home Collection Kit that allowed for the rapid expansion of SARS-CoV-2 testing nationally (EUA 200283, EUA203174) [20, 21]. The Everlywell COVID-19 Test Home Collection Kit was composed of sample registration instructions, sample collection instructions, sample preparation and shipping instructions, nasal swab, saline in a tube, shipping materials, and return labels. Instructions were included to direct the home users on how to appropriately collect the nasal swab specimen and place it in the saline transport tube, how to properly package the specimen, and how to mail the specimen back to the laboratory using the pre-labeled return envelope (or to otherwise arrange for specimen pick-up via courier). Each Home Collection Kit was intended to be returned via overnight courier service at ambient conditions on the same day of or the day following sample collection in accordance with the standards as put forth by the U.S. Center for Disease Control (CDC) and World Health Organization (WHO) for the transport of suspected COVID-19 samples.

Data were included in the study by querying the Everlywell database for all COVID-19 tests from May 19, 2020 to September 21, 2021. All samples included in the analysis were self-collected outside of a healthcare setting and processed at independent partner laboratories operating under the EUA framework. Test results were considered positive or negative for SARS-CoV-2 based on their PCR cycle threshold (Ct) values. All complete test results for the COVID-19 Test Home Collection Kit from those over the age of 18 with associated self-reported symptom information were considered eligible for inclusion in the study. Exclusion criteria included inconclusive PCR results (i.e., those that could not be clearly identified as Positive or Negative based on lab-specific limits of detection), samples with insufficient quantity for PCR testing, samples determined as otherwise untestable by lab operators, and repeat positive PCR results within 90 days of each other.

### Symptoms

Symptoms were self-reported in the test registration portal at the time of kit registration. The database consisted only of individuals reporting symptoms determined to be mild, moderate, or absent. Mild to moderate symptoms included: fever between 100.4 and 102 ºF, new or worsening cough, new or worsening sore throat, flu-like symptoms (chills, runny or stuffy nose, whole body aches, a headache and/or feeling tired), shortness of breath (not limiting ability to speak) or a new loss of taste or smell. Those with severe symptoms (Fever > 102 ºF or a high fever lasting > 48 h, inability to speak in full sentences or do simple activities without shortness of breath, severe coughing spells or coughing up blood, blue face or lips, severe and constant pain or pressure in the chest, extreme lethargy, dizziness, lightheadedness or being too weak to stand, slurred speech or seizures, being unable to stay at home due to being too sick) were redirected to acute care centers and did not proceed with the self-collected sampling. Individuals were considered asymptomatic if they responded “No symptoms or symptoms not listed” on the questionnaire presented at the time of kit registration; otherwise, they were considered symptomatic. Those reporting mild and moderate symptoms were grouped as symptomatic, while those reporting no symptoms or symptoms not listed were considered asymptomatic for the analysis. Post-test symptom development was not queried, preventing categorization of a pre-symptomatic subgroup. The average time to complete the online kit registration form was approximately 4 min.

### Exposures

Data on potential SAR-CoV-2 exposures was captured at the time of kit registration. Individuals selected their potential for exposure from standard Health and Human Services screening questions for having (a) no known exposure, (b) experiencing congregate housing and work situations, (c) known exposure to diagnosed or (d) presumed infected individuals. For analysis, responses were grouped as: “No Known Exposure” (a), “Area Community Spread” (b), and “Known Exposure” (c and d).

### Statistical methods

The primary statistical methods used were estimates of prevalence of SARS-CoV-2 among asymptomatic and symptomatic cases, the total prevalence of the virus in the study population and estimates of relative risk of infection given symptom status overall and stratified by demographic characteristics. Logs were taken for clear visualization of small differences in odds ratios. Ninety-five (95) percent confidence intervals were calculated for each prevalence estimate using Wilson’s score method for a single proportion [22]. Welch’s two-sample t-test was used to compare the distribution of Ct values among asymptomatic and symptomatic cases, along with standard summary statistics. Frequencies of reported exposure types among asymptomatic and symptomatic cases were compared using χ^2^ tests. All statistical tests were conducted in RStudio version 4.0.3 for MacOS Catalina.

## Results

### Demographic characteristics and testing

**Fig. 1:**
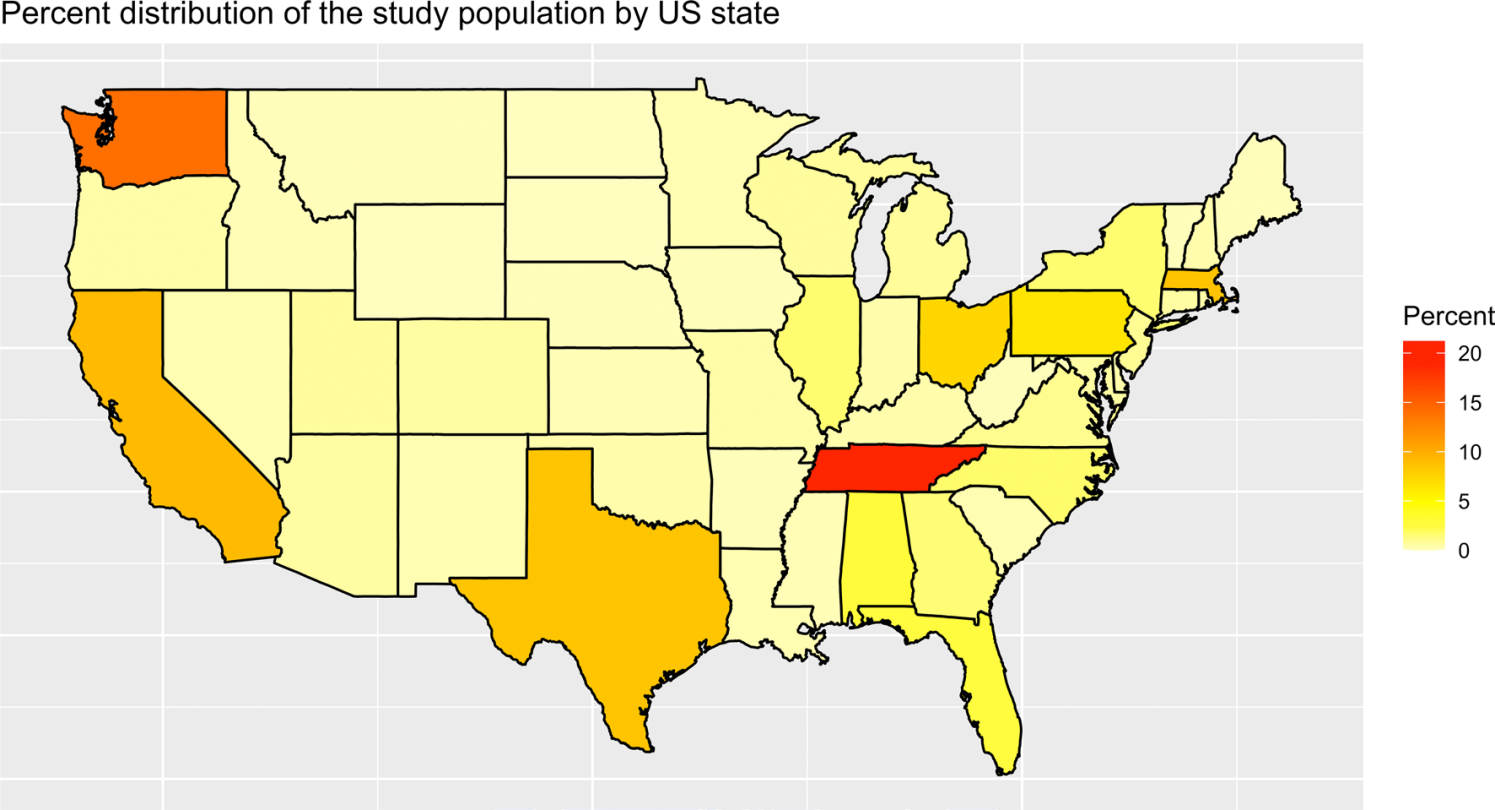
Percent distribution of the study population by state

Data from 282,831 individuals were included in the study. Of these, 199,673 (70.6%) tested for SARS-CoV-2 only once, 40,312 (14.3%) tested twice, and 42,846 (15.1%) tested ≥ 3 times, resulting in a total of 639,332 collection kit results over the 16-month study period (Table 1). Testing and re-testing were electively chosen and individuals’ reasons for testing either once or more than once were not captured. The general demographic distribution of the population evaluated for this study is summarized in Table 2. Just over half the study sample were female (52.7%), 70.8% were under 45 years old, 69.2% were white, and 10.8% were Latino or Hispanic. The geographic distribution of the study population is described by state in Fig. 1.

**Table 1:** Summary of kits, subjects, and test results for the entire study population

	Count
No. of kits	639,332
No. of individuals	282,831
No. of one-time testers	199,673
No. of repeat testers^a^	83,158
No. COVID-19 negative results	613,985
No. COVID-19 positive results	22,152
No. unique COVID-19 positive cases	20,806
No. of reinfections	67
No. inconclusive results	3187
No. kits received but not tested	8

^a^The distribution of the number of repeat tests among included subjects ranged from 2 tests per individual subject to a single subject who tested 81 times

**Table 2:** Demographic characteristics of the study population (N = 282,831)^a^

Group	Value
	N (%)
Sex	
Female	149,044 (52.7%)
Male	133,787 (47.3%)
Age^b^	
18–21 years	53,014 (18.7%)
22–45 years	147,422 (52.1%)
46–65 years	68,464 (24.2%)
66 + years	13,931 (4.9%)
Race	
American Indian or Alaska Native	2003 (0.7%)
Asian	15,398 (5.4%)
Black or African American	27,394 (9.7%)
Native Hawaiian or other Pacific Islander	1127 (0.4%)
White	195,770 (69.2%)
Two or more races	9178 (3.2%)
Other	13,604 (4.8%)
Race Unknown	18,357 (6.5%)
Ethnicity	
Latino or Hispanic	30,498 (10.8%)
Not Latino or Hispanic	218,736 (77.3%)
Ethnicity Unknown	33,597 (11.9%)
Healthcare provider	
Yes	41,439 (14.7%)
No	227,146 (80.3%)
Unknown	14,246 (5.0%)
Risk of severe infection^c^	
High Risk	81,294 (28.7%)
Low Risk	201,537 (71.3%)
Pregnancy status^d^	
Pregnant	2265 (1.5%)
Not pregnant	146,297 (98.2%)
Unknown status	482 (0.3%)

^a^Of these 282,831 individuals, 199,673 only tested once, while 83,158 tested more than once. Sixty-seven (67) individuals (0.02%) were considered reinfected after testing positive twice with > 90 days between the two collection dates

^b^Median age is 35 years; IQR = 27 (22–49)

^c^Risk was determined according to user-reported comorbidities and age

^d^N = 149,044, the number of females in the study population

After applying exclusion criteria, 634,791 test results remained in the final dataset. This number included all samples with a negative PCR result (including repeat tests by the same subject at different time points) and samples with positive PCR results indicating new and independent infections. Only 67 individuals had repeat infections based on this 90-day window.

### SARS-CoV-2 positivity

A total of 20,806 SARS-CoV-2 infections were detected, for an overall positivity rate of 3.28% in the study population. Of these, 10,142 reported no symptoms at the time of kit registration, or 48.75% of all cases. This number corresponds to a 1.73% positivity rate among all asymptomatic individuals. In contrast, among cases reporting symptoms, the positivity rate was 21.61% (Table 3).

**Table 3:** Prevalence of SARS-CoV-2

	Detected	Not detected	Total	Prevalence % (95% CI)	Relative risk
Asymptomatic	10,142	575,292	585,434	1.73% (1.70–1.77)	0.08^a^
Symptomatic	10,664	38,693	49,357	21.61% (21.7–22.7)	12.47^b^
Total	20,806	613,985	634,791^c^	3.28% (3.23–3.32)	NA
Asymptomatic (%)	48.75%	93.70%	92.22%		
Symptomatic (%)	51.25%	6.30%	7.78%		

^a^Relative risk of asymptomatic/symptomatic probabilities of infection

^b^Relative risk of symptomatic/asymptomatic probabilities of infection

^c^The number of total samples evaluated for prevalence and risk analysis is lower than the total study population by removing invalid PCR results (N = 3187) and exclusion of sequential Positive Results within 90 days of each other (N = 1346), for a total of 4533 excluded from this analysis

### Relative risk of positive test result

**Fig. 2:**
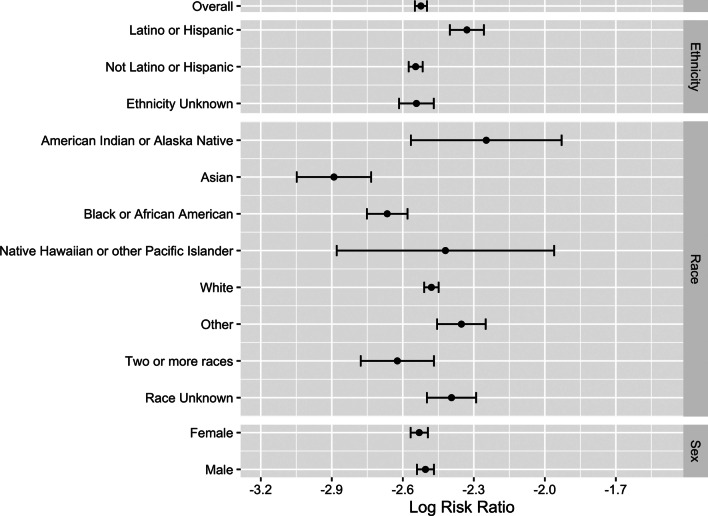
Risk of SARS-CoV-2 positivity in those reporting no symptoms relative to those that reported symptoms. The overall log risk ratio of a positive case given no symptoms to infection with symptoms is − 2.52 (0.080 on a non-log scale). We present these data on a log scale to visualize the relationships among each subcategory more clearly. Error bars are 95% confidence intervals, computed using Wald’s normal approximation and log-transformation

The overall log risk ratio of a positive test result among asymptomatic individuals relative to symptomatic individuals was − 2.52 (raw scale: 0.080, 95% CI, 0.078–0.082; Table 3; Fig. 2), and the log risk ratio in the reverse case was 2.52 (raw scale: 12.47, 95% CI, 12.15–12.79; Table 3). Among all demographic stratifications, the log relative risk of a case without symptoms was significantly less than 0.0 (raw scale: 1.0; Fig. 2).

### Exposures

Of the asymptomatic population that tested positive for SARS-CoV-2, 30.8% reported no known exposure compared to 17.6% among symptomatic individuals (χ^2^ = 308.02, df = 1, p < 2.26e−16; Table 4). A similar percentage of asymptomatic and symptomatic individuals reported exposure through area community spread (e.g., experiencing congregate housing and work situations; 23.5% and 25.2%, respectively; χ^2^ = 18.84, df = 1, p = 1.42e−05), while 45.7% of those without symptoms and 57.1% of those with symptoms reported a direct known exposure (χ^2^ = 197.28, df = 1, p < 2.26e−16).

**Table 4:** Exposures reported by symptom status

	Area community spread^a^	Known exposure^b^	No known exposure^c^	Total
Asymptomatic	2379 (23.5%)	4638 (45.7%)	3125 (30.8%)	10,142 (100%)
Symptomatic	2688 (25.2%)	6093 (57.1%)	1883 (17.6%)	10,664 (100%)
Total	5067 (24.4%)	10,731 (51.6%)	5008 (24.1%)	20,806 (100%)

^a^Individuals experiencing congregate housing and work situations

^b^Individuals indicating they had a known exposure to a diagnosed or presumed infected individual

^c﻿^Individuals indicating they had no known exposure to COVID-19

### Ct values

**Fig. 3:**
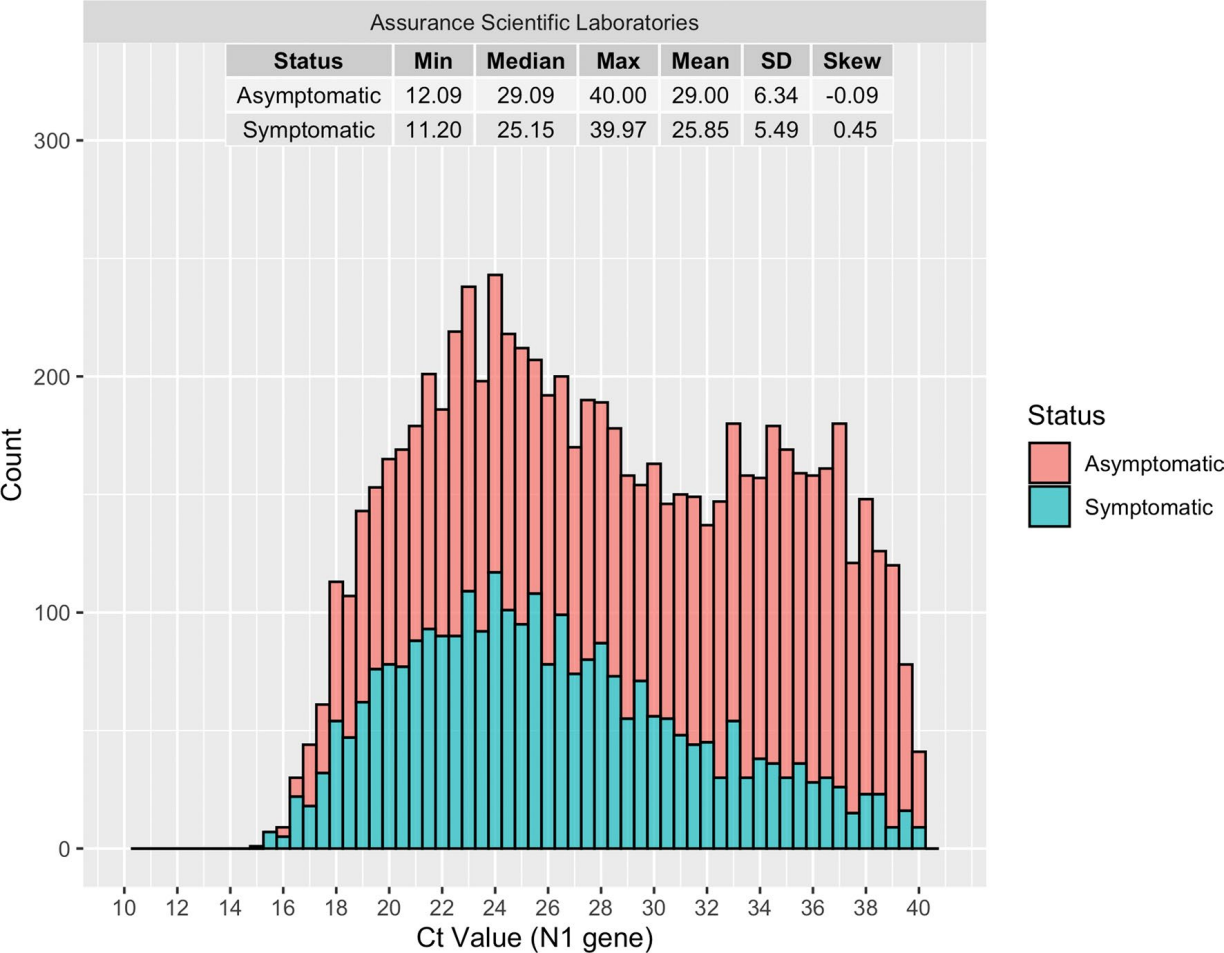
Distributions of Ct values from RT-PCR testing for detection of SARS-CoV-2, stratified by self-reported symptoms. Summary statistics including each distribution’s minimum Ct value, median, maximum, mean, standard deviation (SD), and skewness are presented by status at the top of the figure. Results here are presented for the N1 gene only. Bin widths were set to 0.5 Ct units. N = 7571 positive tests for which Ct values were available from a single partner laboratory, including repeat tests for the same individual within a 90-day window and new infections outside of a 90-day window since the first positive result

The distribution of available Ct values for 7571 positive PCR tests from one of the two partner labs (including repeat positive tests during the time frame of a single infection cycle) stratified by symptom status, are presented in Fig. 3. Ct values are inversely correlated with viral load in a sample, such that a higher viral loads correspond to lower Ct values on a log scale [23, 24]. Notably, the mean Ct value was significantly lower in the symptomatic population than in the asymptomatic population (Welch Two Sample t-test; t = 21.91, p < 2.2e−16). The average Ct of symptomatic compared to asymptomatic cases was 25.85 vs. 29.00, respectively. This observation was supported by the skewness and modality of the relative distributions, with the distribution of Ct values from cases without symptoms being bimodal and slightly left skewed (µ^3^ = − 0.09) while the distribution of Ct values from cases with symptoms was unimodal and slightly right skewed (µ^3^ = 0.45).

## Discussion

This is one of the largest studies to date describing real world at-home sample collection for laboratory based diagnostic PCR testing. Whereas most previous studies focused on single-institutions, samples from smaller populations, and/or healthcare provider-obtained samples, we report over 634,000 representative samples across the United States from real-world, at-home sample collection to estimate the prevalence and relative risk of infection by symptom status [10, 12]. Here we demonstrate that at-home self-collection of anterior nasal swabs is a methodology that should be considered for more broad utilization in overarching COVID-19 testing and pandemic transmission control strategies.

The overall SARS-CoV-2 prevalence of our sample of at-home test takers was lower than national averages during this period (3.28% vs. 7.68%). The lower prevalence may reflect the exclusion of individuals presenting with severe symptoms or those that were seeking symptom management from a healthcare provider at the time of testing. This could also reflect an overall improved accessibility and interest in testing among a wider variety of individuals than the traditional in-person health care worker provided nasopharyngeal swabs.

Importantly, we found that nearly half of those who tested positive reported no symptoms at the time of sample collection, suggesting that among at-home sample collection kit users, asymptomatic carriers are almost as common as individuals with symptoms. This finding is concordant with other reports estimating the percentage of positive cases without symptoms ranges between 20 and 51.9% and highlights the scale to which these estimates correlate to real-world experience [10, 11, 25–28].

In this analysis we cannot definitively distinguish pre-symptomatic cases from those who never developed symptoms, however in a recent meta-analysis it was estimated that ~ 35.1% of all positive cases never show symptoms [26]. In the present population, utilizing this estimate we would predict that 7,302 individuals remained asymptomatic, or 72.0% of all positive cases who reported no symptoms at the time of testing. This estimate is concordant with that of Oran and Topol, who found that nearly three quarters of those who tested positive with no symptoms at the time of testing remained asymptomatic 14 days later [10]. This has important implications for transmission dynamics, as Johansson et al. estimated that 59% of all transmissions may be from asymptomatic individuals, 24% of which are from individuals who never show symptoms [29]. A systematic review and meta-analysis by Buitrago-Garcia et al. reported the secondary transmission rate, or the probability that an infection occurs among susceptible people within a specific group of close contacts, was lower in contacts of people with asymptomatic compared to symptomatic infection (relative risk 0.35, 95% CI 0.10–1.27) [11]. Nonetheless, here we observed a relatively high frequency of individuals without symptoms among people who tested positive, which could help explain the rapid infection rates of COVID-19 despite promotion of social distancing and quarantining of symptomatic individuals. Future work should assess behavior and infection status of close contacts to accurately assess the number of transmissions that could be prevented via a regular at at-home testing model in community and employer settings.

Our analysis of exposures and symptoms found that nearly one third of asymptomatic individuals who tested positive for SARS-CoV-2 reported no known exposures. Currently, the CDC only recommends that asymptomatic individuals get tested if they plan to travel [30] or after a known exposure to COVID-19 [31]. They recommend fully vaccinated individuals should be tested 5–7 days after their last exposure, while those that are not fully vaccinated get tested immediately after a close contact. If the test result is negative for an unvaccinated individual, they should get tested again 5–7 days after their last exposure, or immediately if symptoms develop [31]. However, the CDC also provides guidance that workplace-based testing, particularly for those that are unvaccinated, could identify workers with SARS-CoV-2 infection, and thus help prevent or reduce further transmission [32]. Furthermore, the WHO states testing of asymptomatic individuals is recommended in frequently exposed groups such as health care workers and long-term care facility workers [33]. Indeed, Hellewell et al. estimated that routine testing of asymptomatic individuals every other day could catch up to 94% of cases within 7 days of infection [34]. The primary reason for less frequent testing of asymptomatic individuals is cited as resource limitations [35]. In light of our data, at-home testing may be a critical tool to expand access to testing overall and facilitate increased frequency of testing in asymptomatic individuals without burdening the healthcare system, thereby allowing those who test positive to quarantine and avoid transmitting the virus [36].

This study is one of the largest of its kind to compare the distribution of Ct values between symptomatic and asymptomatic individuals. We found that the mean and median Ct values were significantly lower among symptomatic than asymptomatic cases, indicating that, on average, viral loads are higher in those with symptoms. This finding contrasts with previous studies demonstrating either no difference in the mean Ct value by symptom status, or lower average Ct values from asymptomatic cases; however, their sample sizes were much smaller (n = 48 to 213 infections) and represent single regional sites [37–39]. Conversely, we captured Ct values for 7571 positive tests (including repeat tests for the same infection) across a large geographic region (i.e., United States).

### Limitations

Our real-world analysis has several limitations. The sample population only represented adults > 18 years of age and was biased toward asymptomatic individuals and those with mild to moderate symptoms as those with severe symptoms were redirected to a hospital setting, impacting the generalizability in children or those with more severe symptoms. Because the symptom questionnaire completed at kit registration grouped both those with “no symptoms” and “symptoms not listed” together, it is possible that our asymptomatic subgroup included some individuals with less common COVID-19 symptoms. We were also unable to distinguish individuals who never developed symptoms from those who were pre-symptomatic, which could have inflated the positive case rate for our asymptomatic group. This is a common limitation in epidemiological studies utilizing real world data, as post-test symptom follow-up regarding symptom status is generally not available. Additionally, diagnostic PCR testing has a delayed turnaround of 24–72 h and can continue to detect previous infections beyond the transmissible stage of the virus. Here we utilized a 90-day window to identify independent infections, but persistent presence of SARS-CoV-2 nucleic acid or protein detection for beyond 90-days has been reported [40, 41]. It is possible that some of the identified asymptomatic cases in fact represented previous infections unrelated to this test. However, in a consistent asymptomatic testing program, a positive test from a previous infection would be a known event and not classified as a new infection or require additional quarantine.

Finally, as this is an epidemiological study utilizing real-world data, without confirmatory testing, it is not possible to determine the exact number or rates of false positive or false negative results in the present analysis. The positive and negative percent agreement statistics for each partner laboratory’s assays can be found in their respective EUAs [42–45]. False positive and false negative results may have been present in the dataset due to cross-contamination, presence of PCR inhibitors, sample inadequacy, and/or viral mutations in some cases [46]. Additionally, as the test utilized herein only detects the N1 gene, mutations of this gene (as well as other causes of failed detection) without an alternative gene to amplify may contribute to potential false negatives. Individual results, especially negative results, from home-collected samples should therefore be interpreted in the context of symptoms, exposure profiles, and local outbreak dynamics when managing suspected cases in a clinical setting.

## Conclusions

Routine asymptomatic testing could play an important role to slow the spread of COVID-19 and control the pandemic while reducing burden on the healthcare system. Considering the relative contribution of asymptomatic transmission to the growth of the pandemic, and that we found nearly half of SARS-CoV-2 positive test results were in cases that didn’t report symptoms at the time of testing, we believe at-home collection and mail-in lab tests may be vital in reducing SARS-CoV-2 spread in high-transmission areas. At-home collection kits further serve as an important tool for public engagement in COVID-19 mitigation strategies and as demonstrated here, in epidemiological research. We emphasize the ongoing importance of routine asymptomatic screening through at-home collection methods as new variants of concern arise, and as reports accumulate of waning immunity between 4 and 6 months after vaccination or previous infection.

## Data Availability

The datasets used and/or analysed during the current study are available from the corresponding author on reasonable request.

## Abbreviations

CDC: Center for Disease Control
COVID-19: Coronavirus disease 2019
Ct: Cycle threshold
EUA: Emergency Use Authorization
FDA: US Food and Drug Administration
NAAT: Nucleic acid amplification testing
RT-PCR: Reverse transcriptase polymerase chain reaction
SARS-CoV-2: Severe acute respiratory syndrome coronavirus
U.S.: United States
WHO: World Health Organization
